# One size does not fit all: income-sensitive thresholds for catastrophic health expenditure

**DOI:** 10.1093/heapol/czag013

**Published:** 2026-02-06

**Authors:** Jay Dev Dubey, Dushyant Kumar, Bheemeshwar Reddy A

**Affiliations:** Department of Economics & Finance, Birla Institute of Technology & Science, Pilani Hyderabad Campus Jawahar Nagar, Kapra Mandal Dist., Medchal, Telangana 500 078, India; Department of Economics & Finance, Birla Institute of Technology & Science, Pilani Hyderabad Campus Jawahar Nagar, Kapra Mandal Dist., Medchal, Telangana 500 078, India; Department of Economics & Finance, Birla Institute of Technology & Science, Pilani Hyderabad Campus Jawahar Nagar, Kapra Mandal Dist., Medchal, Telangana 500 078, India

**Keywords:** catastrophic health expenditure, out-of-pocket expenditure, component-specific catastrophic threshold, rank-weighted index, concentration index

## Abstract

This study develops an inverse rank-weighted index (IRWI) to adjust catastrophic thresholds for out-of-pocket expenditure (OOPE) components. The proposed method eliminates the arbitrariness of the existing proportionate approach by ensuring fairness in determining component-specific catastrophic thresholds. It measures the effective expenditure share of each OOPE component while considering the concentration of component-specific spending across household income levels. Using nationally representative household survey data on healthcare consumption from 2017–8, the study estimates catastrophic health expenditure (CHE) at both aggregate and component levels in India under uniform, proportionate, and IRWI thresholds. The findings reveal that the uniform threshold significantly underestimates CHE incidence, whereas component-specific thresholds identify twice as many households that experience CHE. Shifting from the proportionate method thresholds to IRWI thresholds significantly alters CHE estimates. The IRWI approach offers a more reliable framework for integrating component-specific and aggregate CHE assessments. It underscores the need for income-sensitive, component-specific thresholds to accurately quantify financial hardship and prevent underestimating healthcare-related economic burden.

Key messages

- The study proposes a new method to set fair, component-specific thresholds for catastrophic health spending.
- Existing uniform and proportionate methods fail to capture income-based variation and often underestimate or overestimate healthcare-related financial distress.
- The new approach reflects spending patterns more accurately and identifies vulnerable households more effectively.
- Applying the methods to Indian data shows a significant shift in catastrophic health expenditure estimates under the inverse rank-weighted index approach. The financial burden from essential components like medicines rises, while the burden from non-essential items, such as transport, declines.

## Introduction

Out-of-pocket expenditure (OOPE) remains a predominant method of healthcare financing, particularly in low- and middle-income countries (LMICs). Excessive OOPE leads to catastrophic health expenditure (CHE), driving households below the poverty line and perpetuating cycles of economic hardship. CHE often restricts timely and adequate access to healthcare, exacerbating health inequities and undermining efforts to achieve universal health coverage (Haghparast-Bidgoli et al. 2015). Several studies have extensively examined CHE measurement, emphasizing its critical role in assessing financial protection in healthcare (Mohsin et al. 2024).

Conventional CHE measurement captures a situation in which a household’s OOPE for healthcare surpasses a set proportion of their income (Wagstaff and van Doorslaerc 2003).Standard methods of measuring CHE include the budget share and capacity-to-pay approaches. The budget share method defines CHE as OOPE spending that exceeds 10% or 15% of income, while the capacity-to-pay approach sets a 40% threshold based on income after covering basic needs. Methods of measuring CHE face criticism due to inconsistencies in definitions and data limitations. For example, the budget share method does not differentiate healthcare users according to income level. Similarly, the capacity-to-pay approach ignores the distortion caused by healthcare spending on other necessities. This method requires extensive data that is often unavailable in LMICs and defining ‘basic needs’ remains contentious. Both methods often fail to capture long-term financial hardships and access barriers, limiting their effectiveness in thoroughly assessing healthcare affordability and financial distortion (Xu et al. 2003, Nguyen et al. 2023).

A critical yet largely overlooked aspect of CHE measurement is the role of individual OOPE components in driving financial distress. In systems such as LMICs, where fee-for-service models dominate, accessing healthcare involves purchasing complementary yet essential distinct medical goods and services (such as consultations, pharmaceuticals, diagnostics, care units, and other medical goods and services.). The demand for specific healthcare services varies significantly based on ailment type, treatment nature, and cost of services. Unique market dynamics govern these medical products, and several studies have analysed them by measuring demand responsiveness or price elasticity across different healthcare services. Studies show that acute illness, preventive, and emergency services are price inelastic, while pharmaceuticals, speciality care, and elective procedures are price elastic (Duarte 2012, Ellis et al. 2017). Cross-price elasticity influences demand by linking price changes of one healthcare service to changes in demand for another (Deaton 1987). Empirical studies report diverse outcomes, suggesting that inpatient and outpatient services substitute for each other (Ringel et al. 2002, Zhou et al. 2011), while Manning et al. (1987)’s study reveals negative elasticity, indicating complementarity. Other studies, such as Winkelmann (2004), find that rising medicine costs significantly reduce the demand for doctor visits. In addition, Li et al. (2007) show that under price-sharing policies, patients substitute free physician consultations with increased medication purchases.

In LMICs, the fee-for-service model creates a trade-off between medications and linked services, with negative cross-price elasticities dominating due to financial constraints. This insight is crucial in the current context due to the potential for negative cross-price elasticity, driven by the complementary nature of various OOPE components. In such cases, an increase in the price of one medical service can decrease the demand for other related services. This poses a significant risk of compromised healthcare consumption, due to financial catastrophe generated by one or more healthcare components.

Extending traditional methods to component- or service-specific evaluation of CHE incidence presents unique challenges. When specific components of OOPE are disproportionately expensive, households may have limited resources to spend on other necessary healthcare services. Since healthcare consumption relies on a composite of such services, this could mean that even after surpassing the CHE threshold, some healthcare needs remain unmet. This situation equates to denial of care or access barriers. Consequently, the impoverishment caused by CHE, coupled with unmet healthcare needs, is more severe than current measures indicate. A more careful approach requires choosing a component-specific CHE threshold.

Ataguba et al. (2024) provide an innovative method (hereafter referred to as the proportionate method) for determining these thresholds based on the relative expenditure share of each service within the total OOPE. Under this framework, the threshold for a specific component depends on its proportional contribution to overall expenditure. For instance, if pharmaceuticals account for 60% of total OOPE and the overall CHE threshold is set at 10%, the proportionate method would assign 10% of 60% (i.e. 6%) as the CHE threshold for pharmaceuticals. Using a 10% threshold for each component to classify OOPE as catastrophic is overly rigid and causes a misleading estimation of CHE measures. For example, Akazili et al. (2025) showed that applying service-specific thresholds in Ghana uncovered substantial variation in the financial burden across different health services. They further highlight that high-cost services, particularly hospitalisation, drive CHE disproportionately, which would likely be underestimated if uniform thresholds were applied.

The proportionate method addresses underestimation of CHE incidences that arise when component-specific thresholds are unadjusted. However, the rationale for adjusting thresholds based on the expenditure share of a component is not robust. For example, if pharmaceuticals constitute the largest share of OOPE, then the method allocates the highest CHE threshold for it. The issue is that the approach is arbitrary and still prone to underestimate CHE incidence for a crucial OOPE component, such as pharmaceuticals, by allocating a large threshold. The proportionate method overlooks how each component of OOPE impacts different socioeconomic groups. For example, while pharmaceuticals may form a significant portion of overall OOPE, the share might be disproportionately higher among economically disadvantaged groups. Poor households often spend a larger portion of their OOPE on medications compared to other healthcare services. This financial strain is exacerbated by the high and variable costs of medicines, which are often unaffordable for the economically disadvantaged. As medication costs rise, this financial burden increases, especially when combined with non-medical expenses such as transportation (Roy et al. 2012, Selvaraj et al. 2018, Ambade et al. 2022). Adopting a more robust criterion is essential for accurately determining service-specific thresholds.

The paper addresses this gap by introducing a component-specific catastrophic threshold allocation method. It proposes an inverse rank-weighted index (IRWI) (see the Method section) to calculate optimal thresholds for evaluating CHE incidence. The method calculates the effective share of each OOPE component by adjusting for an index that ranks the expenditure share of that component across households. This approach ensures that the contribution of each component reflects variations in spending among households paying for healthcare. The method focuses on identifying vulnerabilities emanating from component-specific OOPE burden distributed across economic subgroups. The method considers how a household’s expenditure share for each component of OOPE varies with income level. For example, higher-income households may have a larger expenditure share for specific components, while lower-income households may spend more on others, such as medicines. This paper introduces a novel approach that extends the proportionate method by incorporating fairness in determining the threshold for the CHE of OOPE components.

A clear understanding of component-wise OOPE is vital for health policies to tackle CHE and provide measurable financial relief to households. Targeted interventions help policymakers focus on high-cost components like medicines and diagnostics, reducing OOPE and maximising the economic impact of health spending to protect vulnerable populations (Kadarpeta et al. 2024). It also enables health systems to prioritise resources effectively, addressing critical health needs while ensuring cost-effectiveness and equity. In resource-limited settings like India, these strategies enable need-based investments in healthcare components (Atun et al. 2010). Need-based public financing can optimise health system efficiency and enhance equity (Farooqui et al. 2022). Evidence shows that many beneficiaries of state-sponsored insurance schemes continue to incur OOPE on medicines and transportation, highlighting critical institutional gaps. Expanding coverage to include outpatient care and other non-hospitalisation costs is essential to achieving comprehensive financial risk protection (Balooni et al. 2012). Medicines, consumables, and human resources constitute a substantial portion of healthcare costs. Shortages of essential medicines in public facilities further exacerbate OOPE, particularly for outpatient care, revealing a pressing need for targeted interventions to improve affordability and accessibility (Prinja et al. 2016).

The study also highlights an important limitation shared by the conventional, proportional, and IRWI methods. These methods only measure the incidence and intensity of CHE and offer no insight into the dispersion of OOPE above the CHE threshold. A comprehensive assessment of financial distress due to OOPE requires an evaluation of severity, which depends not only on the number of households that cross the threshold and to what extent, but also on how these margins are dispersed. For example, many households exceed the threshold by a moderate amount on the medicine component. However, for surgery, only a few cross the specific threshold, but by huge margins. These situations reflect distinct forms of vulnerability and call for different policy responses. Recent literature by Ogwang and Mwabu (2024, 2025) offers a holistic approach to studying CHE by utilising income-poverty measures. These approaches enable the measurement of inequality in CHE payments. The present study builds on this feature to examine how CHE payments vary across different components of healthcare.

In light of this, the study begins with a brief methodological discussion of the conventional and proportionate approaches, followed by a detailed explanation of the proposed IRWI method. The Results section applies the IRWI approach to the Indian context using a large household survey database and compares the findings with conventional and proportionate methods. The sensitivity and stability of estimates based on component-specific thresholds derived from the IRWI approach are evaluated in the Discussion. Finally, in the Conclusion the study’s limitations are outlined and potential areas for future research are identified.

## Method

### Conventional CHE measurement approach

This section briefly revisits the widely used conventional approach to measuring CHE, which relies on a uniform threshold method (Wagstaff and van Doorslaerc 2003). Consider a population of *N* households, where the health expenditure of the $i^{th}$ household is $h_{i}\geq 0$ and its income is $y_{i}$. Since the survey data provides consumption expenditure information of the households, following Deaton (1987) we use this as the proxy for $y_{i}$. Let *z* represent a predetermined catastrophic threshold. If the share of health expenditure in a household’s income exceeds z%, that household is considered to be facing financial catastrophe due to healthcare payments. Assuming $v_{i}=\frac{h_{i}}{y_{i}}$, the conventional approach assigns a value of 1 if $v_{i}>z$ and 0 otherwise, ensuring that households with spending share crossing the catastrophic threshold are identified by a binary variable (equation 1). Consequently, equation (2) estimates the conventional CHE headcount and equation (3) measures the mean positive gap (MPG) or overshoot [or CHE intensity; see Wagstaff and van Doorslaerc (2003) for the definition].


(1) $$\begin{array}{rl} CHE_{i}= & 1\;\mathrm{if}\;v_{i}>z=0\;\mathrm{Otherwise} \end{array}$$

(2) $$H_{z}=\frac{1}{N}\overset{n}{\underset{i=1}{\sum }}\;CHE_{i}$$

(3) $$MPG_{z}=\frac{1}{N}\overset{n}{\underset{i=1}{\sum }}\;\left(v_{i}-z\right)/H_{z}$$

### Component-specific proportionate threshold

Literature addressing the measurement of service- or disease-specific CHE and related indicators often employs the framework outlined in equations (1) and (2). However, Ataguba et al. (2024)’s work proposes that the aggregate share of OOPE components should guide the selection of service-specific thresholds. Let there be K≥2 components within OOPE. Assume $h_{ik}\geq 0$ is the expenditure on the $k^{th}$ healthcare component or service by the $i^{th}$ household. Further, if $S_{k}$ represents the expenditure share of the $k^{th}$ service in total OOPE, then z% of $S_{k}$ should determine the specific threshold or the $k^{th}$ service (denoted by $\alpha_{k}$). Where, *z* is the standard threshold for aggregate expenditure. Equation (4) constitutes the proportionate method for allocating component- or service-specific catastrophic thresholds proposed by Ataguba et al. (2024).


(4) $$\begin{array}{rl} & S_{k}=\frac{\overset{n}{\underset{i=1}{\sum }}\;h_{ik}}{\overset{n}{\underset{i=1}{\sum }}\;h_{i}}\\ & \alpha_{k}=zS_{k}\\ & \overset{K}{\underset{k=1}{\sum }}\;S_{k}=1\;\mathrm{and}\;\overset{K}{\underset{k=1}{\sum }}\;\alpha_{k}=z \end{array}$$

### IRWI threshold

The widespread adoption of the conventional uniform threshold method in CHE analysis is evident from its extensive use in studies addressing specific public health concerns and geographical disparities. Many such studies measure CHE using conventional fixed thresholds for major public health issues, such as obstetrics, HIV, injuries, tuberculosis (TB), cancer, and non-communicable diseases (NCDs). The conventional approach may not address the intricacies of specific public health issues. Communities in different geographic settings experience varying vulnerabilities, often shaped by disease burdens and healthcare infrastructure (de Siqueira Filha et al. 2022). Emerging diseases among heterogeneous population groups may not align with a country’s existing financial protection policies (Haakenstad et al. 2019). In such situations, healthcare demand may lean disproportionately toward specific treatment components. For example, despite Ethiopia’s free TB treatment policy, pre-diagnosis costs imposed significant financial burdens on the patients (Assefa et al. 2024). The component-specific CHE thresholds call forth the importance of analysing the incidence of payment at various stages of healthcare.

A key point in the proportionate approach is that this method assigns a lower catastrophic threshold to a component that makes up a smaller share of total OOPE. This means even a minimal expense can push the care seeker into the catastrophic range, potentially qualifying them for policy support. However, this could create selection bias because demand elasticity varies for different OOPE components across socio-economic groups. To address this, the analysis introduces an alternative method for service-specific thresholds. This method creates a rank-weighted index. It uses the share of the $k^{th}$ component for the $i^{th}$ household’s total healthcare expenditure ($h_{i}$) and considers the relative concentration based on household income levels (per capita). More precisely, assume $s_{ik}=\frac{h_{ik}}{h_{i}}$ is the share of $k^{th}$ component in $h_{i}$ for the $i^{th}$ household such that ${\hat{S}}_{k}$ is the mean of $s_{ik}$.


(5) $${\hat{S}}_{k}=\frac{1}{N}\overset{N}{\underset{i=1}{\sum }}\;s_{ik}=\frac{1}{N}\overset{N}{\underset{i=1}{\sum }}\frac{h_{ik}}{h_{i}}$$

Note that, while $S_{k}$ is the overall share of the $k^{th}$ component in the total expenditure, ${\hat{S}}_{k}$ represents a household-specific average, summarizing how each household allocates its total spending to that component. Therefore, $S_{k}$ gives an aggregate perspective, while ${\hat{S}}_{k}$ accounts for intra-household variations. $S_{k}$ and ${\hat{S}}_{k}$ would be equal only if there is no intra-household variation in the spending of the $k^{th}$ component.

Let $r_{i}$ denote the absolute income rank of the $i^{th}$ household with corresponding relative rank given by $R_{i}=\frac{r_{i}}{N}$. Thus, the household with the lowest per capita income is allocated rank 1 or the relative rank of $\frac{1}{N}$, second lowest is $\frac{2}{N}$ and so on for the $N^{th}$ household $\frac{N}{N}$, such that richest household will have a relative rank of 1. Therefore, following the expression in Kakwani et al. (1997), equation (6) depicts the concentration index (CI) of the ratio $s_{ik}$.


(6) $$CI_{k}=\frac{2}{N{\hat{S}}_{k}}\overset{N}{\underset{i=1}{\sum }}\;s_{ik}R_{i}-1$$

Equation (6) evaluates how the component share $s_{ik}$ is distributed along the income ranking. It relates each household’s share on the $k^{th}$ component to its relative income rank $R_{i}$. Note that $-1\leq CI_{k}\leq 1$ and a positive value of $CI_{k}$ indicates that richer households allocate a larger share of their OOPE to component *k*, while a negative value shows a heavier burden among poorer households. The mean and CI of $s_{ik}$ may interact together to construct the effective mean expenditure share of the $k^{th}$ OOPE component. For this, following Wagstaff and van Doorslaerc (2003) consider $w_{i}=2\frac{N+1-r_{i}}{N};\;\frac{2}{N}\leq w_{i}\leq 2$ as a weighting system on $s_{ik}$ and define the index ${\hat{W}}_{k}$


(7) $${\hat{W}}_{k}=\frac{1}{N}\overset{N}{\underset{i=1}{\sum }}\;w_{i}s_{ik}$$

For large *N* the index ${\hat{W}}_{k}$ can be expressed in terms of equation (8) which follows the properties given in equation (9)


(8) $$\begin{array}{rl} {\hat{W}}_{k}= & \;{\hat{S}}_{k}(1-CI_{k})(\mathrm{see}\;\mathrm{proof}\;\mathrm{in}\;\mathrm{supplementary}\;\mathrm{Appendix}\\ & \;A.1\;\mathrm{in}\;\mathrm{the}\;\mathrm{online}\;\mathrm{supplementary}\;\mathrm{material}.) \end{array}$$

Since, $\overset{K}{\underset{k=1}{\sum }}\;{\hat{S}}_{k}=1\;\mathrm{and}\;\overset{K}{\underset{k=1}{\sum }}\;{\hat{S}}_{k}CI_{k}=0$


(9) $$\begin{array}{rl} & \mathrm{Therefore},\overset{K}{\underset{k=1}{\sum }}\;{\hat{W}}_{k}=1(\mathrm{see}\;\mathrm{proof}\;\mathrm{in}\;\mathrm{supplementary}\;\mathrm{Appendix}\\ & A.2\;\mathrm{in}\;\mathrm{the}\;\mathrm{online}\;\mathrm{supplementary}\;\mathrm{material}.) \end{array}$$

A negative CI ($CI_{k}$) for the *k*th component indicates that higher values of $s_{ik}$ are concentrated among lower-income groups, inflating ${\hat{W}}_{k}$. This suggests that the issue of a high share in the total OOPE by the $k^{th}$ component among poorer households is worse than it appears. Conversely, a positive CI deflates ${\hat{W}}_{k}$, balancing the overstatement of high share problems among the affluent. Thus, ${\hat{W}}_{k}$ represents the effective mean expenditure share of the $k^{th}$ component. The expenditure share of different healthcare components affects households differently depending on income. For two households with equal shares of expenditure for a given component, the poorer household experiences a heavier burden. For instance, if the community average for medicine expenditure is 30%, but poorer households spend 50% and wealthier households only 10%, the aggregate average (30%) misrepresents the actual distribution of the medical expenditure share across households with varying incomes. Therefore, relying on an effective rate provides a more precise view of expenditure heterogeneity across income classes.

Note that ${\hat{W}}_{k}$ will be highest for the component of OOPE with the lowest (or most negative) CI. In the rank-weighted approach, using a component-specific threshold is logical if it allocates the smallest weight to this component. The idea is that components disproportionately affecting poorer households should have a lower threshold. This approach ensures they are more effectively included under the catastrophic expenditure measure. A normalised transformation of ${\hat{W}}_{k}$ according to equation (10) yields inverse rank weight ($I_{k}$) to determine the final adjusted component-specific threshold (${\hat{\alpha }}_{k}$) for the $k^{th}$ component. Table E.1 in supplementary Appendix E (see online supplementary material) illustrates a hypothetical example for the application of the IRWI approach.


(10) $$\begin{array}{rl} I_{k} & =\frac{1-{\hat{W}}_{k}}{\overset{K}{\underset{k=1}{\sum }}\;(1-{\hat{W}}_{k})}\\ I_{k} & =\frac{1-{\hat{W}}_{k}}{K-1}\\ {\hat{\alpha }}_{k} & =zI_{k} \end{array}$$

### CHE inequality: Foster–Greer–Thorbecke approach

The present analysis, together with the IRWI-based component-specific threshold, follows the framework developed by Ogwang and Mwabu (2024), who adapt the Foster–Greer–Thorbecke (FGT) class of poverty measures [Foster et al. (1984)] to study the CHE beyond incidence and intensity. Foster et al. (1984) introduced a rank-independent measure of income poverty that satisfies the fundamental axioms of a good poverty measure (Aristondo 2018), thereby expressing the FGT poverty index in terms of the incidence, intensity, and inequality of income poverty. These desirable properties also remain valid in the context of CHE. Therefore, the FGT-type CHE measure is a comprehensive index that reflects the combined influence of incidence, intensity, and inequality in a single framework.

Assume there are $H_{k}$ households experiencing CHE for the $k^{th}$ component, with the respective IRWI threshold. The FGT-type CHE formulation in the notation adopted till now is given by equation (11) with λ≥0 as the CHE aversion parameter.


(11) $$CHE_{k}(\lambda )=\frac{1}{N}\overset{N}{\underset{i=1}{\sum }}\;(C_{ik}{)}^{\lambda },\;C_{ik}=\{\begin{array}{cc} \frac{v_{ik}-{\hat{\alpha }}_{k}}{{\hat{\alpha }}_{k}} & \mathrm{if}\;v_{ik}>{\hat{\alpha }}_{k},\\ 0 & \mathrm{otherwise} \end{array}$$

In equation (11), setting λ=0 reduces the index to the headcount, as in the conventional approach. When λ=1, the FGT-type CHE index captures the normalised average intensity of catastrophic payments. Finally, with λ=2, equation (11) provides the FGT-type CHE index that represents the full spectrum of OPPE-related financial distress. To explain this, define $\Gamma_{k}(\lambda )$ as the average FGT score for any given λ>0 (equation 12). Further, equation (13) illustrates the multiplicative decomposition property of the FGT-type CHE measure as the incidence-weighted average FGT score. The representation enables expressing $CHE_{k}(\lambda )$ for λ=2 in equation (14), in terms of incidence, intensity, and inequality (see supplementary Appendix D in the online supplementary material).

Suppose


(12) $$\Gamma_{k}(\lambda )=\frac{1}{H_{k}}\overset{H_{k}}{\underset{i=1}{\sum }}\;(C_{ik}{)}^{\lambda }$$

then


(13) $$CHE_{k}(\lambda )=\left(\frac{H_{k}}{N}\right)\times \Gamma_{k}(\lambda )$$

also


(14) $$CHE_{k}(2)=\left(\frac{H_{k}}{N}\right)\times [\Gamma_{k}^{2}(1)+\sigma^{2}(C_{ik})]$$

## Results

### The case of India

India faces a high burden of OOPE due to weak public healthcare systems and reliance on private providers (Haakenstad et al. 2022, Prinja et al. 2022). This dependence escalates OOPE and increases the risk of CHE (Mishra and Mohanty 2019). Studies highlight a consistent rise in healthcare costs since the 1980s, worsening the financial strain on households (Gumber et al. 2017). Between 2004 and 2014, mean OOPE increased, with wealthier households spending more on inpatient care and poorer households on outpatient services. CHE for inpatient care rose significantly during this period, underscoring growing inequality (Akhtar et al. 2020).

Recent evidence indicates that while India has experienced a decline in OOPE, as reported in the National Health Accounts (2018–19), financial distress persists. Nearly 16% of households rely on distress financing to cover hospitalisation-related OOPE. Moreover, outpatient care imposes a disproportionate burden, contributing to a poverty rate of 15%, significantly higher than the 10.7% linked to hospitalisation (Nanda and Sharma 2023). CHE varies significantly depending on the type of ailment.

NCDs such as cardiovascular diseases, cancer, and diabetes, along with injuries incur exceptionally high treatment costs, with CHE rates of ∼50% for these conditions (Yadav et al. 2021). Healthcare components such as medications, diagnostics, surgeries, and hospital stays contribute differently to OOPE, depending on the type of ailment. Chronic diseases like diabetes and hypertension lead to consistent expenses due to the need for regular medication and diagnostics. On the other hand, acute ailments, including cancer and cardiovascular conditions, often result in high one-time costs from surgeries and extended hospital stays.

Rural households face additional challenges. Limited access to healthcare facilities often forces patients to travel long distances. Transport costs and food and lodging for attendants significantly increase OOPE for rural populations. These non-medical costs can be a significant burden, especially when combined with the high expenses of private care (Kastor and Mohanty 2018, Katyal 2021).

### Data

The analysis utilises data from the Social Consumption of Health Survey for 2017–18, conducted during the 75th round of the household survey by the National Statistical Office (NSO) of India. This survey includes 113 823 households and 555 353 individuals, with data from 93 925 inpatient and 43 240 outpatient cases. The survey includes socioeconomic and demographic information of sampled households. Additionally, the survey collects information on the type of healthcare provider, such as public or private, enrolment in government insurance schemes, access to private insurance, household members with chronic illnesses or permanent disabilities, and female members who are currently pregnant. This information allows for a detailed assessment of household healthcare consumption patterns. Further, it also enables extensive multidimensional empirical research on healthcare consumption patterns across India.

The database offers detailed OOPE information for hospitalisation and outpatient care. Allowing horizontal disaggregation, the survey records expenses on distinct service components, including doctor’s fees, medication, diagnostics, bed charges, and other expenditures borne by the households that are directly or indirectly attributed to OOPE for healthcare (see Table 1 for details). OPPE for inpatient care includes a marginal subset of cases buying package components for care. Cases involving bundled service packages—predominantly used by wealthier households—are excluded due to insufficient expense breakdowns. For households unable to report expenditure break-ups due to recall issues or inadequate hospital documentation at discharge, the survey reports only aggregate OOPE. The present analysis of inpatient OOPE also excludes such cases. As a result, the analysis includes 72 356 households using inpatient services and 29 723 households using outpatient services.

**Table 1:** Healthcare services constituting OOPE.

*k*	OOPE components—NSO 2017–18	
	Inpatient	Outpatient
1	Doctor’s/surgeon’s fee	Doctor’s/surgeon’s fee
2	Medicines	Medicines: AYUSH^a^
3	Diagnostic tests	Medicines
4	Bed charges	Diagnostic tests
5	Other medical expenses	Other medical expenses
6	Transport for patient	Transport for patient
7	Other non-medical expenses	Other non-medical expenses

^a^AYUSH stands for Ayurveda, Yoga & Naturopathy, Unani, Siddha, and Homeopathy.

### Incidence of consumption and mean OOPE

Table 2 reveals that nearly all households (99.8%) incur OOPE for inpatient care, paying for at least one of the seven OOPE components. Compared to standard medical services, more households report above zero expenses on non-standard items, such as attendant charges, physiotherapy, and personal medical appliances (see supplementary Appendix F in the online supplementary material for a detailed definition of components). Public inpatient facility users may avail themselves of a few services free of charge, such as consultation, bed, diagnostics, and pharmaceutical services. However, those receiving treatment in private institutions must pay for such services. Note that therapeutic and consumable costs are borne directly by either type of user. Therefore, the incidence of consumption of other medical items is significantly higher than that of standard medical items. Non-medical expenses, such as registration fees and ambulance services, are incurred by both public and private users, resulting in a high incidence of consumption for this component. For outpatient care, the trend differs; households predominantly spend on pharmaceuticals and fees, with less being spent on diagnostics and non-medical costs. A slightly higher proportion of outpatient cases incur zero expenses than inpatient cases.

**Table 2:** Descriptive statistics.

Components	Incidence^a^	Mean	Std. error	$\mu \pm 1.96\frac{\sigma }{\sqrt{n}}$		*N*
	(%)	(INR)		L	U	
Hospitalisation cases						
Doctor’s/surgeon’s fee	46.1	7709	108	7498	7921	32876
Medicines	79.3	6150	70	6012	6287	59360
Diagnostic tests	62.7	3322	37	3249	3394	48161
Bed charges	44.6	4777	62	4655	4898	32471
Other medical expenses	94.6	1560	13	1535	1585	43004
Transport for patient	92.1	767	6	755	780	67508
Other non-medical expenses	58.3	2850	55	2743	2958	68307
Total OOPE: inpatient	99.8	16507	152	16209	16804	72178
Outpatient cases						
Doctor’s/surgeon’s fee	43.7	6187	188	5819	6555	12125
Medicines: AYUSH^b^	6.9	15039	642	13780	16298	2021
Medicines	81.4	15704	255	15204	16203	24217
Diagnostic tests	17.9	14092	426	13257	14927	5915
Other medical expenses	8.6	9920	887	8180	11659	2969
Transport for patient	56.1	2880	46	2789	2970	17138
Other non-medical expenses	35.2	3282	74	3137	3427	10489
Total OOPE: outpatient	95.6	23705	366	22987	24422	28220
Consumption expenditure	100	112855	242	112380	113330	113823

^a^Incidence refers to the proportion of households making a positive payment in a given category. The mean expenditure for each component is measured in Indian Rupees (INR). $\mu \pm 1.96\frac{\sigma }{\sqrt{n}}$ is the confidence interval of the mean expenditure and *N* is the corresponding sample size.

^b^AYUSH stands for Ayurveda, Yoga & Naturopathy, Unani, Siddha, and Homeopathy.

The mean positive yearly OOPE varies significantly across components, with standard medical expenses generally higher than non-standard ones for inpatient and outpatient care. Among inpatient components, doctor’s/surgeon’s fees rank highest. However, the standard deviation suggests uneven payment distribution across households. This may imply the possibility of a disproportionate burden on certain socioeconomic groups. For outpatient care, pharmacy expenses dominate OOPE, followed by diagnostics charges. Other medical expenses, though lower in frequency, are notably higher for outpateint care. This may be due to patients with chronic conditions facing uncovered recurring costs. The significant variability in expenses underscores the financial strain at the component level. These findings highlight the need to explore how individual components contribute to CHE and deepen the understanding of financial distress among households.

### Component-specific CHE

The conventional unadjusted threshold (Wagstaff and van Doorslaerc 2003) method severely underestimates financial distress caused by OOPE at the component level compared to the proportionate (Ataguba et al. 2024) method threshold (Table 3). The conventional method requires households to spend >10% of total consumption expenditure on a component to qualify as CHE. This strict condition includes only a few severely affected households. In contrast, the proportionate method adjusts thresholds based on a component’s share of total health expenditure. The method identifies financial distress more fairly and addresses the underestimation issue. In this method, as observed medicines (inpatient) account for 29.6% of health expenses, the specific CHE threshold becomes 2.96%, showing that even smaller (compared to the conventional case) expenses on certain services can be catastrophic. For inpatient care, the conventional method shows more CHE cases for doctor’s fees than for diagnostics. The proportionate method approach reverses this ranking. Similar reversals occur between bed charges and doctor’s/surgeon’s fees. Non-medical costs like transport affect a few households under the conventional method but >5% under the proportionate method. For outpatient care, these gaps are even wider.

**Table 3:** Comparison of CHE incidence and intensity: conventional and proportionate methods^a^.

Components	Conventional method				$S_{k}$	$\alpha_{k}$	Proportionate method			
	$H_{10}$	$CI_{10}$	$MPG_{10}$	$CI_{M_{10}}$			$H_{\alpha_{k}}$	$CI_{\alpha_{k}}$	$MPG_{\alpha_{k}}$	$CI_{M_{\alpha_{k}}}$
Hospitalisation cases										
Doctor’s/surgeon’s fee	1.05	−0.028	19.41	−0.071	21.5	2.15	3.16	0.081	10.88	−0.126
Medicines	1.47	−0.115	17.78	−0.023	29.6	2.96	4.43	−0.059	9.81	−0.058
Diagnostic tests	0.53	−0.058	13.65	−0.038	12.9	1.29	3.93	0.060	4.95	−0.099
Bed charges	0.48	−0.115	12.75	0.010	12.6	1.26	4.39	−0.014	4.21	−0.055
Other medical expenses	0.37	−0.091	18.23	0.019	10.1	1.01	3.42	−0.018	4.60	−0.047
Transport for patient	0.07	−0.332	6.68	−0.036	4.3	0.43	5.54	−0.158	1.19	−0.092
Other non-medical expenses	0.24	−0.204	12.47	0.011	9.0	0.90	5.44	−0.105	2.44	−0.071
Outpatient cases										
Doctor’s/surgeon’s fee	1.32	−0.099	12.21	0.055	11.9	1.19	9.52	0.057	5.35	−0.088
Medicines: AYUSH	0.60	−0.023	24.19	0.015	4.6	0.46	1.68	0.009	14.55	−0.023
Medicines	8.24	−0.053	23.64	−0.096	56.4	5.64	12.20	−0.016	19.61	−0.111
Diagnostic tests	1.45	−0.079	23.92	−0.059	11.1	1.11	4.13	0.057	13.84	−0.152
Other medical expenses	0.37	0.097	29.29	−0.019	3.7	0.37	1.95	0.028	9.69	0.017
Transport for patient	0.73	−0.279	12.91	−0.004	7.1	0.71	10.79	−0.019	3.29	−0.171
Other non-medical expenses	0.57	−0.281	12.48	0.104	5.1	0.51	7.68	−0.057	3.70	−0.123

^a^All indicators are expressed as percentages (%). H denotes the catastrophic headcount, with the corresponding concentration index denoted by CI. MPG represents the mean positive gap, and its CI is denoted by $CI_{M}$. The suffix indicates the catastrophic threshold used. The conventional method uses 10% threshold for each component. $S_{k}$ denotes the expenditure share of the $k^{\mathrm{th}\;}$ component, with the proportional catastrophic threshold given by $\alpha_{k}$.

Note that the proportionate method leads to a downward adjustment of the conventional threshold. The lower cut-off increases the incidence of CHE and raises the magnitude of overshoots (intensity) for any given component. Following the definition of *MPG* (equation 3) and the findings in Table 3, the proportionate threshold produces a faster rise in incidence than in overshoot. Hence, for each component, $MPG_{10}>MPG_{\alpha_{k}}$. Table 3 also reveals that CHE intensity overestimation in the conventional method is linked directly to headcount underestimation. The linkage is governed by how the threshold shift under the proportionate adjustment aligns with the income distribution of households.

Households enter the catastrophic net by making large absolute healthcare payments or having such low consumption expenditures that even minimal OOPE can convert into catastrophic incidence. Large absolute payments apply to only a small number of households. The high conventional threshold is inclined to capture the impoverished households within the scope of catastrophic expenditure. The conventional method, as shown in Table 3, yields negative CIs for the headcount across both inpatient and outpatient components. However, this leaves out households with slightly better incomes, who still experience financial distortions from healthcare payments. Such households face catastrophic spending unofficially but may be ruled out from receiving adequate policy attention. The proportionate method addresses this issue. In this approach, some components exhibit small positive CIs ($CI_{k}$) of CHE incidence ($H_{\alpha_{k}}$). In contrast, others show large negative indices, thereby broadening the identification of distressed households.

However, this pattern reverses for the *MPG* when transitioning from the conventional to the proportional threshold. The $MPG_{10}$ estimate is influenced by households who are able to bear a high OOPE burden, resulting in a skewed measure of CHE intensity. The proportional adjustment of the threshold ensures that the intensity measure $MPG_{\alpha_{k}}$ better represents the overshoot among poorer households while also accounting for those who require policy attention but do not qualify under the rigid conventional catastrophic threshold.

### IRWI-based CHE estimates

The proportionate method reduces underestimation generated by the conventional approach but needs a more robust justification for allocating component-level thresholds. For instance, the inpatient transport component has a low aggregate expenditure share (4.3%) in total OOPE, leading to a small proportionate threshold (0.43%). The small threshold classifies a large number of households as experiencing CHE. As a result, the catastrophic headcount for transport becomes the highest among all inpatient OOPE components under the proportionate method (see Table 3). This pattern indicates an elevated risk of false positives or overestimation of CHE incidence in the proportionate approach. Notably, transport exhibits the lowest mean expenditure and the smallest household expenditure share, yet it generates the highest CHE incidence across components. Therefore, accounting for its distribution across income groups remains essential (see Table 2 and Table 3). The IRWI method considers the effective mean expenditure share for transport relative to total OOPE. Note that the observed mean expenditure share ${\hat{S}}_{k}=0.125$ leans significantly toward poorer households ($CI_{k}=-0.096$). Therefore, the resulting effective mean expenditure share increases accordingly (${\hat{W}}_{k}=0.137$). Hence, the IRWI approach reconciles the inpatient transport threshold from 0.43% to 1.44%. This adjustment effectively excludes false positives, or households incorrectly classified into the catastrophic net for the transport component despite having the ability to afford it, and reduces the transport-related headcount from 5.44% to 1.68% (see Table 4).

**Table 4:** Rank-weighted component-wise catastrophic threshold and headcount ratio.

Components^a^	$\underset{k}{\hat{S}}$	$CI_{k}$	$\underset{k}{\hat{W}}$	$I_{k}$	$\underset{k}{\hat{\alpha }}$	Hα^k
Hospitalisation cases						
Doctor’s/surgeon’s fee	0.100	0.183	0.081	0.153	1.53%	3.77%
Medicines	0.283	−0.037	0.294	0.118	1.18%	7.61%
Diagnostic tests	0.107	0.057	0.101	0.150	1.50%	3.53%
Bed charges	0.073	0.190	0.059	0.157	1.57%	3.91%
Other medical expenses	0.082	0.006	0.081	0.153	1.53%	2.48%
Transport for patient	0.125	−0.096	0.137	0.144	1.44%	1.68%
Other non-medical expenses	0.231	−0.070	0.247	0.125	1.25%	4.05%
Outpatient cases						
Doctor’s/surgeon’s fee	0.108	0.084	0.099	0.150	1.50%	8.86%
Medicines: AYUSH^b^	0.045	−0.071	0.048	0.159	1.59%	1.50%
Medicines	0.605	0.002	0.604	0.066	0.66%	20.1%
Diagnostic tests	0.044	0.054	0.041	0.160	1.60%	3.93%
Other medical expenses	0.017	−0.010	0.017	0.164	1.64%	1.44%
Transport for patient	0.112	−0.015	0.113	0.148	1.48%	6.99%
Other non-medical expenses	0.069	−0.106	0.077	0.154	1.54%	4.81%

^a^ ${\hat{S}}_{k}$ denotes the mean household expenditure share for the $k^{\mathrm{th}\;}$ component. $CI_{k}$ is the corresponding concentration index of household expenditure share. ${\hat{W}}_{k}={\hat{S}}_{k}(1-CI_{k})$ denotes the effective expenditure share, with the corresponding inverse rank-weighted index (IRWI) given by $I_{k}$, and ${\hat{\alpha }}_{k}$ represents the catastrophic threshold applied to obtain the corresponding catastrophic headcount $H_{{\hat{\alpha }}_{k}}$ for the $k^{\mathrm{th}\;}$ component.

^b^AYUSH stands for Ayurveda, Yoga & Naturopathy, Unani, Siddha, and Homeopathy.

In contrast, medicine expenditure for institutionalised patients shows a high mean expenditure share, with high shares marginally concentrated among poorer households. However, the catastrophic threshold shifts downwards from 2.96% to 1.18%, raising the CHE headcount significantly compared to the proportionate method (from 4.43% to 7.61%). For outpatient medicines, this adjustment is even starker. In outpatient care for medicine expenditure, the headcount estimates see a significant shift (from 12.2% to 20.7%). The headcount estimates under the IRWI approach align better with India’s healthcare system, where medicine expenses significantly escalate OOPE. Spending on drugs and medicines overwhelmingly dominates OOPE and contributes significantly to the CHE situation (Sangar et al. 2022). Although medicine costs are often smaller than other expenses, they can accumulate rapidly, particularly for individuals with chronic conditions (Saksena et al. 2010).

The relationship between the proportionate and IRWI methods is not one-sided. The IRWI adjustment may yield higher or lower component-specific thresholds than the proportionate method, depending on a household’s expenditure share for a given component relative to its position in the income distribution. The catastrophic headcount adjusts accordingly. The rank-weighted approach addresses biases in threshold allocation by the proportionate method, offering a more consistent framework that reflects the component-specific CHE situation.

### CHE comparison at the aggregate level

The conventional approach significantly underestimates aggregate CHE. For both inpatient and outpatient OOPE, component-specific thresholds identify twice as many households under CHE as the conventional method. Both proportionate threshold adjustment and the IRWI method address the highly underestimated aggregate CHE obtained by the conventional method, but with different degrees. The aggregate CHE incidence for inpatient healthcare is slightly higher in the proportionate method. This pattern contrasts with outpatient cases, where the IRWI method captures a significantly larger share of households under CHE. The findings are consistent with India’s public health system trends. However, these results also put emphasis on the methodological differences and the need for careful threshold selection to ensure accurate and nuanced CHE assessment (Table 5).

**Table 5:** Catastrophic headcount at the aggregate level^a^.

Item	Threshold	$\frac{H}{N}(\text{\%})$
Aggregate IP OOPE	Conventional (10%)	4.57
At least one IP components is catastrophic	Proportionate method	9.48
	IRWI method	9.19
Aggregate OP OOPE	Conventional (10%)	12.12
At least one OP components is catastrophic	Proportionate method	19.24
	IRWI method	22.64

^a^IP = Inpatient or hospitalisation; OP = outpatient; OOPE = out-of-pocket expenditure; IRWI = inverse rank-weighted index. $\frac{H}{N}=$ Catastrophic headcount.

### FGT-type CHE measures

The FGT-type CHE approach provides a unified framework to measure CHE’s incidence, intensity, and inequality through a single formulation for various aversion levels. Both intensity and inequality measures are based on a normalised threshold, allowing the measures to be comparable across components. Γ(1) represents the average normalised healthcare spending gap, capturing the mean by which expenditures exceed the catastrophic threshold. Γ(2), the squared average normalised healthcare spending measures the fluctuation in healthcare payment by the CHE-encountering households. A higher squared coefficient of variation of normalised health expenditure relative to the catastrophic threshold indicates greater inequality among households experiencing CHE.

Table 6 reports the estimates of the CHE parameters within the FGT-type CHE measure framework. A complete profile of CHE is obtained by evaluating the index at the aversion parameters λ=0,1, and 2. The FGT-type CHE framework captures the severity of overshoots by progressively weighting each component’s relative OOPE margin above its corresponding IRWI threshold. An additional advantage of combining the FGT approach with the IRWI-based component-specific thresholds is that it preserves comparability across healthcare components despite differences in their respective thresholds. For instance, in the inpatient doctor’s/surgeon’s fee component, households that face CHE spend about 6.3 times the threshold. This level exceeds the overshoot in the inpatient medicine component, where affected households spend roughly 6 times the threshold. However, the population-level intensity measure, $CHE_{k}(1)$, shows a different pattern. The average overshoot reaches 23.9% of the threshold in doctors’/surgeons’ fees. It rises to 45.7% in the medicine component.

**Table 6:** FGT-based incidence, intensity and inequality measures.

Components^a^	${\hat{\alpha }}_{k}$	$CHE_{k}(\lambda )$			$\Gamma_{k}(\lambda )$		$\Gamma_{k}^{2}(1)$	$\sigma^{2}(v_{ik}^{*})$
		λ=0	λ=1	λ=2	λ=1	λ=2		
Hospitalisation cases								
Doctor’s/surgeon’s fee	1.53%	0.038	0.239	10.2	6.3	271.9	40.1	231.8
Medicines	1.18%	0.076	0.457	20.0	6.0	263.0	36.1	227.0
Diagnostic tests	1.57%	0.035	0.118	2.9	3.3	81.7	11.1	70.6
Bed charges	1.50%	0.039	0.117	2.0	3.0	51.7	8.9	42.7
Other medical expenses	1.53%	0.025	0.093	3.1	3.7	123.4	14.0	109.4
Transport for patient	1.44%	0.017	0.024	0.1	1.4	8.8	2.1	6.8
Other non-medical expenses	1.25%	0.041	0.092	1.3	2.3	31.6	5.2	26.4
Outpatient cases								
Doctor’s/surgeon’s fee	1.50%	0.089	0.321	10.9	3.6	122.9	13.1	109.8
Medicines: AYUSH^b^	1.59%	0.015	0.143	5.8	9.5	384.7	89.9	294.9
Medicines	0.66%	0.201	4.820	1307.2	24.0	6512.5	576.8	5935.9
Diagnostic tests	1.60%	0.039	0.346	27.6	8.8	703.0	77.3	625.8
Other medical expenses	1.64%	0.014	0.103	14.7	7.1	1020.0	50.5	970.0
Transport for patient	1.48%	0.070	0.196	3.0	2.8	42.9	7.8	35.0
Other non-medical expenses	1.54%	0.048	0.143	2.4	3.0	49.3	8.8	40.5

^a^ ${\hat{\alpha }}_{k}$ denotes the inverse rank-weighted index (IRWI) based catastrophic threshold for the $k^{\mathrm{th}\;}$ component. CHEk(λ) is the Foster–Greer–Thorbeceke (FGT)-type CHE measure for the $k^{\mathrm{th}}$ component. $\Gamma_{k}(\lambda )$ is the average FGT score associated with the aversion parameter λ. Also, $v_{\mathrm{ik}}^{*}=\frac{v_{\mathrm{ik}}}{{\hat{\alpha }}_{k}}$ denotes the standardised OOPE share, with the corresponding variance denoted by $\sigma^{2}(v_{\mathrm{ik}}^{*})$.

^b^AYUSH stands for Ayurveda, Yoga & Naturopathy, Unani, Siddha, and Homeopathy.

An additional strength of this framework lies in equation (14), which expresses $CHE_{k}(\lambda )$ at λ=2 in terms of incidence, intensity, and inequality. This depiction of $CHE_{k}(2)$ decomposes the contributions of both the overshoot magnitude and its dispersion across households. For example, in the inpatient doctor’s/surgeon’s fee category, the intensity ($\Gamma_{k}(1)$) accounts for only 15% of the total value of $CHE_{k}(2)$. The remaining 85% arises from the variance of intensity [$\sigma^{2}(C_{ik})$]. This indicates that households exceeding the threshold do so by a substantial margin. This pattern appears across all inpatient and outpatient components. The pattern is most pronounced in outpatient medicine, where inequality dominates the CHE profile. Such pronounced inequality raises serious concerns, as it may reflect the high cost of medicines for chronic and severe conditions where patients rely on continuous medication.

### Comparison of CHE pattern from 71st round of NSO

The analysis incorporates data from the 71st round of the NSO survey (2014–15) to understand the CHE pattern over time and ensure consistency of the component-specific methods [(tables for the 71st round are provided in supplementary Appendix G (supplementary Tables G.1–G.5), see online supplementary material]. CHE incidence was considerably higher in 2014–15 for inpatient and outpatient care.

In the 71st round, the inpatient CHE headcount was 6.63% under the conventional method. By comparison, the proportionate method estimated it at 12.39%, while the IRWI approach reported 12.07%. In the 75th round, all three methods showed significant reductions.

The results indicate that higher income inequality and healthcare consumption contributed to greater financial distress in the 71st round. The National Health Account (2018–19) shows that the share of OOPE on health in India is ∼52% of current health expenditure. Compared to previous OOPE share of 63% in current health expenditure during 2016–17 (NHA). The reduced CHE is accounted for by such improvement.

The pattern of CHE measures across components and care types remains consistent in both survey rounds. For example, medicine component accounts for the highest CHE proportion in both rounds and for inpatient and outpatient care. However, the magnitude was severely high in the 71st round. The IRWI headcount for outpatient medicine expenses decreased from 26.38% to 20.7%, and for inpatient doctors’ fees, from 5.4% to 3.77%, from the 71st round to 75th round.

The expenditure share structure for inpatient and outpatient care remained broadly unchanged in the two rounds. As a result, the component-specific thresholds in the proportionate method were nearly identical in both rounds. Despite this, CHE headcounts were significantly higher in the 71st round. For instance, the CHE threshold for the medicine component in inpatient care remained constant, yet headcount in the 71st round was 2% higher compared to the 75th round. Conversely, the doctor’s/surgeon’s fee threshold (inpatient) was higher in the 71st round, yet it pulled 1.37% more households into CHE. These differences were even higher for outpatient components.

The IRWI thresholds across the components were almost similar over the NSO rounds. However, in the former survey, some major components, such as doctors/surgeons and medicine (inpatient), depict a rise in mean expenditure share with reduced CI magnitude. The situation shows the elevated financial burden, which disproportionately impacts poorer households during the 71st round. The IRWI thresholds capture the situation more prominently. Similar to the 75th round estimates, the proposed approach rationally redistributed proportionate method CHE incidence across the components. However, in this case, the magnitude of headcounts remained excessively high at the component level (stemming from high OOPE burden and inequality) compared to the 75th round. In high OOPE and inequality scenarios, the IRWI provides a more comprehensive measure of OOPE-induced financial distress.

## Discussion

Our novel IRWI approach advances CHE measurement methods and derives component- or service-specific thresholds for assessing CHE. IRWI accounts for the effect of income distribution on the allocation of household resources to specific healthcare components or services. The proposed method emphasizes that the share of expenditure for specific healthcare components at the household level aligns with household income. The previously introduced approach by Ataguba et al. (2024) mitigates the underestimation caused by conventional methods but provides insufficient justification for threshold allocation. The proportionate method assumes that components with higher expenditure shares inherently contribute less to CHE incidence. Consequently, the method’s higher threshold allocation to high-expenditure share components may misrepresent their actual financial impact, leading to potential misclassification of CHE cases. Notably, a component’s share in total OOPE alone does not determine its likelihood of pushing households into financial distress or CHE. Such limitations result in inconsistent estimations. For instance, the headcount estimation for a non-standard component, such as patient transport, is higher than that for standard OOPE components like medicines. This discrepancy arises from the lower threshold assigned to components such as patient transport expenses, driven by its smaller share in total OOPE, which fails to provide an adequate rationale.

The IRWI method calibrates proportionate method estimates and demonstrates that standard components, such as medicine expenses, have a more significant catastrophic impact compared to non-standard items like patient transport. The IRWI method offers a distinct advantage and identifies households at elevated risk more effectively over conventional or proportionate methods. Poorer households operate under a binding budget constraint, with spending decisions restricted to the boundary of affordability. An increase in expenditure on one medical component often necessitates reducing spending on another. For example, high pharmaceutical costs may force a low-income household to opt for a lower-quality alternative when purchasing orthopaedic support equipment. Wealthier households may treat various healthcare components as complementary, utilising a broad range of services without significant financial strain. In contrast, poorer households often face trade-offs, prioritising essential medical expenses over less-critical services. Thus, many of the poorer households experience unmet healthcare needs or forgo care altogether (Ergo et al. 2019, Sharma et al. 2023).

Studies from LMICs show that fee-for-service systems often generate CHE through specific cost components. In India, Haakenstad et al. (2019) shows that eliminating private drug purchases would avert a significant amount of CHE. Bigio et al. (2023) shows that poor access, limited availability, and high prices of diagnostics delay detection and raise OOPE, which increases CHE due to unpredictable expenses. Surgical care also imposes a severe financial strain. Shrime et al. 2015 report that medical and non-medical costs associated with surgery, such as transport, lodging, and food, fall heavily on poorer households. Component- or service-specific CHE is pervasive in sub-Saharan countries such as Ghana and Ethiopia, as noted by Akazili et al. (2025), which also highlights that this phenomenon extends to many high-income countries, including the USA, China, and Portugal, and is not limited to LMICs.

Different studies show that component or service-specific CHE, such as expenditure on medicines, diagnostics, or surgical care, is widespread in LMICs (Shrime et al. 2015, Haakenstad et al. 2022, Bigio et al. 2023). This is particularly concerning because our findings demonstrate that conventional thresholds systematically underestimate CHE at both aggregate and component levels. The conventional CHE approach remains the standard metric for monitoring progress toward financial protection under Sustainable Development Goal 3.8.2 (Wagstaff et al. 2018). However, its application to specific healthcare components can yield misleading estimates and may result in the exclusion of vulnerable households from policy attention. These limitations highlight the need to broaden the current definition of financial protection within the Sustainable Development Goal framework by incorporating component-specific thresholds that more accurately capture the heterogeneous risks of CHE across healthcare services, especially among poorer households.

### Sensitivity of IRWI thresholds

It is important to understand the sensitivity of the thresholds and resulting CHE measures at the component level in the presence of an outlier in one or more components. Proportionate method thresholds would be highly sensitive to such cases. The presence of an outlier for an important component, e.g. medicine, would elevate the expenditure share and consequently elevate the threshold of the component. This will result in CHE underestimation of medicine due to a comparatively large threshold. This rise in share and threshold would cause a smaller threshold of other components due to reduced expenditure share, causing inappropriate selection of households under CHE concerning these components. These may be non-standard OOPE components with lower penetration in total expenditure.

The IRWI method can mitigate this issue to a significant extent. The reason is that the CI of component share (equation 6) maintains stability in CHE accounting, by absorbing the impact of the outliers. To understand this, consider a case where each household has an equal expenditure share for the $k^{th}$ component i.e. ∀i,j (i≠j), $s_{ik}=s_{\;jk}={\bar{s}}_{k}$. Under this assumption, the aggregate expenditure share and mean household expenditure share are equal, and the CI of the $k^{th}$ component equals zero (equation 13). Consequently the expression for inverse-rank weight in equation (10) can be expressed in terms of aggregate expenditure share of the $k^{th}$ component. Equation (14) enables us to examine the change in $I_{k}$ when all households allocate an equal but high (or very high) share to the $k^{th}$ component. $I_{k}$ and $S_{k}$ are negatively related, and a change in $S_{k}$ leads to a less than one-to-one change in $I_{k}$. The rate of change is constant and depends upon the total number of OOPE components. The final impact on $\alpha_{k}$ and ${\hat{\alpha }}_{k}$ (the catastrophic threshold of the affected and other OOPE components) also depends on *K*. The simplified expression for $I_{k}$ allows for a straightforward derivation of the elasticity of $I_{k}$ ($\varepsilon_{k}$) with respect to $S_{k}$, showing that it depends only on the aggregate expenditure share ($S_{k}$).


$$CI_{k}=0;\;{\bar{s}}_{k}=S_{k}=\hat{S_{k}}=\hat{W_{k}}$$

(16) $$\begin{array}{rl} I_{k} & =\frac{1-S_{k}}{K-1}\\ \frac{dI_{k}}{dS_{k}} & =\frac{-1}{K-1}\\ \varepsilon_{k} & =-\frac{S_{k}}{1-S_{k}} \end{array}$$

In the presence of an outlier, if $S_{k}$ increases (or decreases) and approaches 1 (or 0), the IRWI threshold adjusts downward (upward). To illustrate, assume a six-component world (K=6 in equation 16) where the share of the $k^{th}$ component shifts from $S_{k}=0.1\;\mathrm{to}\;0.9$. The resulting change in catastrophic thresholds $\alpha_{k}$ increases by 8% whereas ${\hat{\alpha }}_{k}$ decreases by 1.6%. Note that for any level of $S_{k}$ the ${\hat{\alpha }}_{k}$ is already significantly small in this example; the response to an outlier by readjusting downwards provides the scope of broadening the CHE net rather than leaving out many outliers as is the case due to inflated $\alpha_{k}$ (larger catastrophic threshold generates underestimated CHE estimates). IRWI tends to have a reciprocal response when the expenditure share of the component is extremely high, affecting poorer households’ healthcare consumption. Alternatively, $\varepsilon_{k}$ shows the differential mechanism of absorbing the shock by IWRI in the case of an extreme value of the component share. At $S_{k}=0.5$, a 1% increase in $S_{k}$ leads to a decrease in $I_{k}$ in the same proportion. However, in the lower range of $S_{k}$, a similar rise allows a smaller decrease in $I_{k}$, but it decreases rapidly for ranges close to one. (ε=0.12 at $S_{k}=0.1$ but ε=9 when $S_{k}=0.9$). Hence, under the assumption (equation 15), if the $k^{th}$ component depicts extreme values of expenditure share, the IRWI catastrophic threshold may reconcile the effect of outliers by stabilizing the thresholds of all the components.

In the previous example, suppose the expenditure share of the $k^{th}$ component is no longer uniform across households ($CI_{k}\neq 0$). Instead, assume the $i^{\prime th}$ household’s expenditure increases significantly, creating an outlier situation. More precisely, from ${\bar{s}}_{k}$, the revised OOPE share for the $i^{\prime th}$ household becomes ${\bar{s}}_{k}+\theta$. The additional expenditure share, *θ*, affects $S_{k}$ and ${\hat{S}}_{k}$. Note that for a certain range of *θ* it can be shown that $S_{k}\geq {\hat{S}}_{k}$. Therefore it is also possible to find *Θ* such that $S_{k}={\hat{S}}_{k}+\Theta$ (see proof in supplementary Appendix B in the online supplementary material for the detailed derivation of the range for *θ*). For further simplification we assume $\delta =(1-CI_{k})$ which allows equation (16) to be re-written as equation (17).

Since, $-1\leq CI_{k}\leq 1$ therefore, 0≤δ≤2


(17) $$\begin{array}{rl} & I_{k}=\frac{1-\delta (S_{k}+\Theta )}{K-1}\\ & \frac{dI_{k}}{dS_{k}}=\frac{-\delta }{K-1}\left[\frac{H}{h_{i^{\prime }}N}\right]\\ & \varepsilon_{k}=\frac{-\delta S_{k}}{1-\delta (S_{k}+\Theta )}\left[\frac{H}{h_{i^{\prime }}N}\right] \end{array}$$

Equation (17) requires $S_{k}+\Theta <\frac{1}{2}$ to ensure $I_{k}>0$ and $\varepsilon_{k}<0$. This condition is necessary for the numerator in the expression for $I_{k}$ (or the denominator in $\varepsilon_{k}$) to remain positive. Given that all components account for <50% of OOPE share in this analysis, the condition is always satisfied in the present context (see proof in supplementary Appendix C in the online supplementary material). In this case too, $I_{k}$ and $S_{k}$ remain negatively related. However, the rate of change depends on *δ*, which is positive but may be greater or smaller than unity, depending on the shift in $CI_{k}$ from its initial value of zero. Suppose the $i^{\prime th}$ household belongs to the poorer group; then, the CI becomes negative, making *δ* greater than one. Conversely, if the $i^{\prime th}$ household is wealthier, *δ* falls below one. Thus, any increase in $S_{k}$ reduces $I_{k}$ faster for poorer households. The resulting ${\hat{\alpha }}_{k}$ is comparatively smaller if the extreme value of $S_{k}$ is concentrated among poorer households. The reciprocal response to an outlier in IRWI is more pronounced when a poorer household experiences an extreme expenditure share. Consider the corner cases where an outlier in $S_{k}$ affects the poorest households ($CI_{k}=-1$). In this case, δ=2, causing $I_{k}$ to decrease twice as fast as in the previous illustration, making ${\hat{\alpha }}_{k}$ extremely small. Conversely, if $S_{k}$ affects the wealthiest households ($CI_{k}=1$), then δ=0, implying no change in ${\hat{\alpha }}_{k}$ in response to the rise in $S_{k}$.

In general $I_{k}$ is increasing in $CI_{k}$, if the outlier exists among poorer households, then the threshold for that component will reduce further, accounting for more households under CHE. In contrast, if an outlier occurs in a wealthy household with a magnitude large enough to push $CI_{k}$ close to one, the thresholds may even rise, causing CHE underestimation. Due to mean expenditure share and CI interplay, the impact will be lower, even when the IRWI threshold increases due to the outlier, compared to the proportionate method. Suppose the component with an outlier is a standard medical item (surgery or medicine), and the household facing it is poor. The overestimation due to the lower threshold would favor the poorer households more by extending the CHE counts. In contrast, if a wealthy household is paying abruptly for a component that is among the non-standard medical expenditures and may not require a similar policy focus, then the underestimation of CHE would probably be less welfare-hurting.

### Stability in IRWI: a simulation framework

The sensitivity analysis was further extended by including a simulation exercise in the dataset. This simulation examines how randomly invoked outliers affect the stability of thresholds derived from both methods. The process begins by identifying the poorest 10% of households based on income rank. From this subset, 10% of households are randomly selected, and their inpatient medicine expenditure is inflated 100 times. Total household expenditure is adjusted accordingly. Component-wise thresholds are then computed using both methods. A parallel exercise is conducted for the top 10% of households according to their income earners. This process is repeated 500 times to capture threshold fluctuations. The volatility in the proportionate method and the relative stability of IRWI thresholds are assessed.

**Figure 1:**
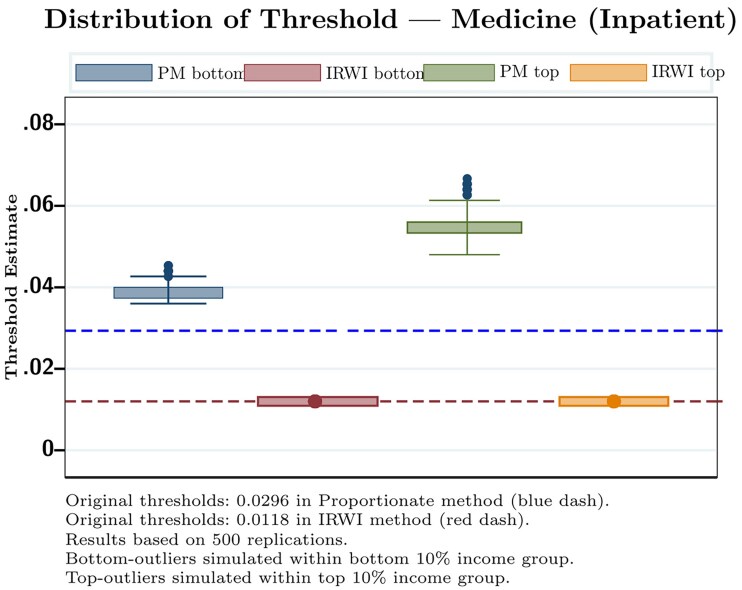
Box plot illustrating threshold movement due to outlier (*Source: Author´s calculation*) .

Figure 1 presents the impact of outliers on inpatient medicine expenditure thresholds. For the poorest households, the proportionate method threshold fluctuates between 0.035 and 0.045, with a mean of 0.039. In contrast, the IRWI threshold remains stable at 0.012. A similar trend appears in the top income group. The proportionate threshold varies between 0.048 and 0.066, while the IRWI threshold remains nearly constant at 0.012. Compared to pre-simulation values (0.030 for proportionate and 0.012 for IRWI), the proportionate method shows considerable volatility. The IRWI approach, however, remains robust. These results confirm that the IRWI method effectively mitigates distortions from extreme values. The method ensures a more stable threshold estimation, making it a reliable approach for measuring CHE.

### Robustness check

Ensuring the robustness of our findings is crucial, particularly when using the IRWI method, which relies on the inequality of component-level expenditure shares across households. IRWI adjusts the catastrophic threshold based on the CI of the component’s expenditure share. However, the standard CI has limitations when applied to bounded variables like expenditure shares, which range between 0 and 1. The traditional CI is also mean-dependent, leading to potential biases in inequality comparisons. A CI of 0.3 for a variable with a mean of 0.2 may reflect stronger inequality than the same CI for a mean of 0.5, but this distinction remains obscured without normalisation (Ataguba 2022).

A robust correction is achieved through the Wagstaff method, which normalizes the CI by dividing it by ($1-{\hat{S}}_{k}$), ensuring more stable comparisons (Wagstaff 2005). Similarly, the Erreygers correction scales the CI by multiplying it by $4{\hat{S}}_{k}(1-{\hat{S}}_{k})$, maintaining sensitivity to inequality across different levels of expenditure share (Erreygers 2009). Applying these corrections ensures that CI remains independent of the mean, preventing artificial reductions when CHE incidence is high. This is particularly important as IRWI also derives thresholds based on mean values.

Given this dependency, the stability of CHE estimates under IRWI has been assessed by applying these corrections. This allows us to evaluate how robust the estimates remain when adjusting for potential biases in the inequality measure. The revised CHE estimates, based on the corrected IRWI thresholds, are presented to verify consistency and reliability.

The robustness check confirms that aggregate CHE estimates remain stable across methods. Inpatient estimates show minor variations, while outpatient estimates remain unchanged. At the component level, IRWI-type thresholds, after CI correction, remain broadly consistent, with only slight variations. For inpatient care, the medicine component lowers the IRWI threshold slightly due to minimal CI changes under Wagstaff correction, while the Erreygers-adjusted threshold remains unchanged. In outpatient care, minor adjustments appear in doctor’s/surgeon’s fees under Erreygers correction and in other non-medical expenses under Wagstaff correction. These adjustments correct bias, ensuring comparability. The findings confirm that methodological refinements preserve ranking and threshold stability (Table 7).

**Table 7:** Robustness check: Wagstaff and Erreygers corrected IRWI threshold.

Components^a^	${\hat{S}}_{k}$	IRWI			Wagstaff correction			Erreygers correction		
		$CI_{k}$	${\hat{\alpha }}_{k}$	$H_{{\hat{\alpha }}_{k}}$	$CI_{k}$	${\hat{\alpha }}_{k}$	$H_{{\hat{\alpha }}_{k}}$	$CI_{k}$	${\hat{\alpha }}_{k}$	$H_{{\hat{\alpha }}_{k}}$
Hospitalisation Cases										
Doctor’s/surgeon’s fee	0.100	0.183	1.53	3.77	0.203	1.53	3.77	0.073	1.51	3.79
Medicines	0.283	−0.037	1.18	7.61	−0.052	1.17	7.62	−0.042	1.18	7.62
Diagnostic tests	0.107	0.057	1.50	3.53	0.064	1.50	3.53	0.024	1.49	3.57
Bed charges	0.073	0.190	1.57	3.91	0.205	1.57	3.91	0.055	1.55	3.92
Other medical expenses	0.082	0.006	1.53	2.48	0.007	1.53	2.48	0.002	1.53	2.48
Transport for Patient	0.125	−0.096	1.44	1.68	−0.109	1.44	1.68	−0.048	1.45	1.68
Other non-medical expenses	0.231	−0.070	1.25	4.05	−0.091	1.25	4.17	−0.065	1.26	4.05
Aggregate CHE-inpatient				9.19			9.24			9.20
Outpatient cases										
Doctor’s/surgeon’s fee	0.108	0.084	1.50	8.86	0.094	1.50	8.86	0.036	1.49	8.91
Medicines: AYUSH^b^	0.045	−0.071	1.59	1.50	−0.074	1.59	1.50	−0.013	1.59	1.50
Medicines	0.605	0.002	0.66	20.07	0.004	0.66	20.07	0.004	0.66	20.07
Diagnostic tests	0.044	0.054	1.60	3.93	0.057	1.60	3.93	0.009	1.59	3.93
Other medical expenses	0.017	−0.010	1.64	1.44	−0.011	1.64	1.44	−0.001	1.64	1.44
Transport for patient	0.112	−0.015	1.48	6.99	−0.017	1.48	6.99	−0.007	1.48	6.99
Other non-medical expenses	0.069	−0.106	1.54	4.81	−0.114	1.54	4.86	−0.029	1.55	4.81
Aggregate CHE-outpatient				22.64			22.64			22.64

^a^All indicators are expressed as percentages (%). ${\hat{S}}_{k}$ denotes the mean household expenditure share for the $k^{\mathrm{th}\;}$ component, while $CI_{k},\;{\hat{\alpha }}_{k}$, and $H_{{\hat{\alpha }}_{k}}$ represent the corresponding concentration index of the individual share, the inverse rank weighted index (IWRI)-based catastrophic threshold, and the catastrophic headcount, respectively.

^b^AYUSH stands for Ayurveda, Yoga & Naturopathy, Unani, Siddha, and Homeopathy.

## Conclusion

The IRWI method presented in this study offers an intuitive approach for deriving catastrophic thresholds specific to each OOPE component. The approach determines thresholds using the expenditure share of each component within a household’s total OOPE and the household’s rank by income, and addresses the issues pertaining to the proportionate method. The integration of the CI for the expenditure share of OOPE components ensures a rational basis for threshold adjustment. The refined threshold allocation by IRWI between standard and non-standard OOPE components leads to substantial changes in CHE estimates. Unlike the proportionate method, IRWI estimates indicate that standard components, such as medicines, contribute more to catastrophic burdens than non-standard items like transport.

Despite these advancements, the study acknowledges limitations. In pay-for-service models of healthcare access, it is rational to assume that the OOPE progresses with the severity of illness. More severe conditions typically demand complex interventions, prolonged care, and multiple services, exerting different financial pressures on OOPE components. An evolved threshold selection approach should account for this variability and classify ailments, such as NCDs, injuries, or acute conditions. Provider preferences, rural–urban disparities, insurance access, and coping mechanisms can also influence threshold selection. Future research could address these issues and adopt more advanced analytical methods, incorporating the price and income elasticity of the OOPE component to provide a more robust and rational catastrophic threshold of the OOPE components.

A natural extension of this work involves constructing a multidimensional CHE index. This will allow the study to fully exploit the disaggregated healthcare expenditure information, coupled with the more refined component-specific IRWI thresholds derived in this work. In this regard, Ogwang and Mwabu (2025) suggest adopting the multidimensional Watts poverty index for measuring multidimensional CHE as suggested in the work of Chakravarty et al. (2008) and Ogwang (2022). Another approach involves applying the dual-cutoff framework proposed by Alkire and Foster (2011a, 2011b). A multidimensional CHE index enriches analysis by revealing the extent to which each healthcare component contributes to the overall catastrophic incidence, intensity, and inequality. In addition, the framework provides a separate intensity of catastrophic spending by counting the number of overshoot cases across all households and expenditure categories relative to the total possible cases.

Several studies identify household income, insurance status, chronic illness, type of healthcare service (inpatient or outpatient), provider type (public or private), and demographic factors as key determinants of overall CHE (Doshmangir et al. 2020). Disaggregating these determinants by expenditure component may reveal distinct and heterogeneous effects across component-specific catastrophic thresholds. Examining these patterns would provide valuable insights and offer directions for further research.

## Supplementary Material

czag013_Supplementary_Data

## Data Availability

The data used in this study was collected by the National Statistical Office (NSO), Government of India, and is freely available in the public domain.(Please visit https://microdata.gov.in/NADA/index.php/catalog/152).

## Reflexivity statement

All authors are male researchers based at a university in India, a low- and middle-income country. Their diverse expertise spans multiple fields of economics, enriching the study’s multidisciplinary approach. One author specialises in health economics, mathematical modeling, and the economics of inequality, bringing critical insight into health policy’s financial and social dimensions. Another author contributes extensive experience in microeconomics and mathematical modeling, supporting rigorous theoretical and quantitative analysis. One of the authors offers expertise in empirical methods and development economics, enhancing the study’s grounding in real-world economic contexts. The authors acknowledge their positionality within an LMIC setting, which informs their understanding of the economic challenges faced by populations similar to India and frames their interpretation of results.
