# Supplementary material for: One size does not fit all: income-sensitive thresholds for catastrophic health expenditure
Source: Health Policy Plan. 2026 Feb 6;41(4):696–711. doi: 10.1093/heapol/czag013 (PMC13089428; doi:10.1093/heapol/czag013)
Supplement: czag013_Supplementary_Data [file czag013_supplementary_data.pdf]

Appendix A Methodological Details

Appendix A.1 Effective expenditure share

The Proof for Equation 8 follows straight from Wagstaff & van Doorslaerc (2003)

$$
\begin{aligned}
\hat{W}_k &= \frac{1}{N}\sum_{i=1}^{N} w_i s_{ik} \\
&= \frac{2}{N}\sum_{i=1}^{N}\left[\frac{N+1-r_i}{N}\right] s_{ik} \\
&= \frac{2}{N}\sum_{i=1}^{N}\left[1+\frac{1}{N}-R_i\right] s_{ik} \\
&= \frac{2}{N}\sum_{i=1}^{N} s_{ik} - \frac{2}{N}\sum_{i=1}^{N} s_{ik}R_i + \frac{2}{N}\sum_{i=1}^{N}\frac{s_{ik}}{N} \\
&= 2\hat{S}_k - \frac{2}{N}\sum_{i=1}^{N} s_{ik}R_i + 2\frac{1}{N}\left(\sum_{i=1}^{N} s_{ik}\right)\frac{1}{N} \\
&= \hat{S}_k - \left[\frac{2}{N}\sum_{i=1}^{N} s_{ik}R_i - \hat{S}_k\right] + \frac{2\hat{S}_k}{N} \\
&= \hat{S}_k - \hat{S}_k\left[\frac{2}{N\hat{S}_k}\sum_{i=1}^{N} s_{ik}R_i - 1\right] + \frac{2\hat{S}_k}{N} \\
&= \hat{S}_k - \hat{S}_k CI_k + \frac{2\hat{S}_k}{N}
\end{aligned}
$$

For large N , i.e. if $N \to \infty$ $\frac{1}{N} \to 0$

$$
\text{Therefore,} \quad \frac{2\hat{S}_k}{N} = 0
$$

$$
\hat{W}_k = \hat{S}_k(1 - CI_k) \tag{A.1}
$$

Appendix A.2 Weighting property of $\hat{W}_k$

Note that, for ith household

$$\sum_{k=1}^{K} s_{ik} = 1$$

$$\text{Since,} \quad \hat{S}_k = \frac{1}{N}\sum_{i=1}^{N} s_{ik}$$

$$\text{Therefore,} \quad \sum_{i=1}^{K} \hat{S}_k = \frac{1}{N}\sum_{i=1}^{K}\sum_{i=1}^{N} s_{ik}$$

$$\sum_{i=1}^{K} \hat{S}_k = \frac{1}{N}\sum_{i=1}^{N}\left(\sum_{i=1}^{K} s_{ik}\right)$$

$$\sum_{i=1}^{K} \hat{S}_k = \frac{1}{N}\sum_{i=1}^{N}(1)$$

$$\sum_{i=1}^{K} \hat{S}_k = 1 \tag{A.2}$$

$$\text{Since,} \quad CI_k = \frac{2}{N\hat{S}_k}\sum_{i=1}^{N} s_{ik}R_i - 1$$

$$\text{Therefore,} \quad \hat{S}_k CI_k = \frac{2}{N}\sum_{i=1}^{N} s_{ik}R_i - \hat{S}_k$$

$$\begin{aligned}
\sum_{i=1}^{K} \hat{S}_k CI_k &= \frac{2}{N}\sum_{i=1}^{K}\sum_{i=1}^{N} s_{ik}R_i - \sum_{i=1}^{K}\hat{S}_k \\
&= \frac{2}{N}\sum_{i=1}^{N}\left(\sum_{i=1}^{K} s_{ik}\right)R_i - 1 \\
&= \frac{2}{N}\sum_{i=1}^{N} R_i - 1 \\
&= \frac{2}{N}\left[\frac{1}{N} + \frac{2}{N} + ... + \frac{N}{N}\right] - 1 \\
&= \frac{2}{N}\left[\frac{1}{N}\frac{N(N+1)}{2}\right] - 1 \\
&= \left[\frac{N+1}{N}\right] - 1 = \frac{1}{N}
\end{aligned}$$

For large N , i.e. if $N \to \infty$ $\frac{1}{N} \to 0$

$$\text{Hence} \quad \sum_{i=1}^{K} \hat{S}_k CI_k = 0 \tag{A.3}$$

Appendix B Sensitivity analysis

Initially, for the kth component, every household's expenditure share is $\bar{s}_k$, implying that $\bar{s}_k = S_k = \hat{S}_k$.

$$\frac{\sum_{i=1}^{N} h_{ik}}{\sum_{i=1}^{N} h_i} = \bar{s}_k$$

$$\text{therefore,} \sum_{i=1}^{N} h_{ik} = \bar{s}_k H$$

$$\text{where} \quad H = \sum_{i=1}^{N} h_i$$

Now, consider an outlier scenario where the expenditure share of the i'^{th} household increases by $\theta \geq 0$. Therefore, the new expenditure share for the kth component becomes $\bar{s} + \theta$ for household $i^{'}$. Since only the allocation to the kth component increases for household $i^{'}$, while its total out-of-pocket expenditure (OOPE) remains unchanged. Hence the aggregate OOPE (H) will also remain unchanged despite the change due to θ. However, θ would affect aggregate expenditure on k^{th} component and therefore it would affect S_k and $\hat{S}_k$. Let the initial expenditure of this household on component k be $h_{i'k}$, and its total expenditure be $h_{i'}$, so that $h_{i'k} = \bar{s}_k h_{i'}$. However, due to θ the increased expenditure on k^{th} component by household $i^{'}$ in the new scenario, say $\hat{h}_{i'k}$ is such that

$$s_{i'k} = \bar{s}_k + \theta,$$

$$\hat{h}_{i'k} = (\bar{s}_k + \theta) h_{i'}$$

The revised expression for S_k is given by Equation B.1 and for $\hat{S}_k$ is given by Equation B.2

$$
\begin{aligned}
S_k &= \frac{\sum_{i \neq i'}^{N} h_{ik} + \hat{h}_{i'k}}{H} \\
&= \frac{\sum_{i \neq i'}^{N} h_{ik} + (\bar{s}_k + \theta) h_{i'}}{H} \\
&= \frac{\sum_{i \neq i'}^{N} h_{ik} + \bar{s}_k h_{i'} + \theta h_{i'}}{H} \\
&= \frac{\sum_{i=1}^{N} h_{ik} + \theta h_{i'}}{H} \\
&= \frac{\bar{s}_k H + \theta h_{i'}}{H} \\
S_k &= \bar{s} + \frac{\theta h_{i'}}{H}
\end{aligned} \tag{B.1}
$$

$$
\begin{aligned}
\hat{S}_k &= \frac{1}{N}[(N-1)\bar{s} + (\bar{s} + \theta)] \\
\hat{S}_k &= \bar{s} + \frac{\theta}{N}
\end{aligned} \tag{B.2}
$$

Now, computing the difference $\hat{S}_k - S_k$ post raising the share for household i' from $\bar{s}$ to $\bar{s} + \theta$.

$$
\begin{aligned}
\hat{S}_k - S_k &= \left(\bar{s} + \frac{\theta}{N}\right) - \left(\bar{s} + \frac{\theta h_{i'}}{H}\right) \\
&= \theta \left(\frac{1}{N} - \frac{h_{i'}}{H}\right)
\end{aligned} \tag{B.3}
$$

$$\text{By assuming} \quad \Theta = \theta\left(\frac{1}{N} - \frac{h_{i'}}{H}\right)$$

$$\hat{S}_k = S_k + \Theta \tag{B.4}$$

Note that both S_k and $\hat{S}_k$ are the function of θ therefore to assess the change in I_k (in Equation 15) due to a change in S_k must account for the change in $\hat{S}_k$ due to change in S_k. For this taking partial derivatives of S_k and $\hat{S}_k$ with respect to θ in Equation B.1 and B.2

$$\frac{\partial S_k}{\partial \theta} = \frac{h_{i'}}{H} \quad \text{and} \quad \frac{\partial \hat{S}_k}{\partial \theta} = \frac{1}{N} \tag{B.5}$$

Hence,

$$\begin{aligned}\frac{d\hat{S}_k}{dS_k} &= \frac{\partial \hat{S}_k/\partial \theta}{\partial S_k/\partial \theta} \\ &= \frac{1/N}{h_{i'}/H} = \frac{H}{Nh_{i'}}\end{aligned}$$

Therefore, we differentiate Equation 15 to obtain change in inverse rank due to change in expenditure of share of the kth component and the corresponding elasticity (Equation B.7 and B.7).

$$I_k = \frac{1 - \delta(S_k + \Theta)}{K - 1}$$

$$
\begin{aligned}
\frac{dI_k}{dS_k} &= \frac{dI_k}{d\hat{S}_k}\frac{d\hat{S}_k}{dS_k} \\
&= \frac{-\delta}{K-1}\left[\frac{H}{h_{i'}N}\right]
\end{aligned} \tag{B.6}
$$

and,

$$
\begin{aligned}
\varepsilon_k &= -\frac{dI_k}{dS_k}\frac{S_k}{I_k} \\
&= -\frac{dI_k}{d\hat{S}_k}\frac{d\hat{S}_k}{dS_k}\frac{S_k}{I_k} \\
&= \frac{-\delta}{K-1}\left[\frac{H}{h_{i'}N}\right]\frac{S_k}{\frac{1-\delta(S_k+\Theta)}{K-1}} \\
&= \frac{-\delta S_k}{1-\delta(S_k+\Theta)}\left[\frac{H}{h_{i'}N}\right]
\end{aligned} \tag{B.7}
$$

Appendix C Feasible conditions for Equation 17

Note that since $\frac{d\hat{S}_k}{dS_k} \geq 0$, therefore feabile condition for $I_k \geq 0$ and $\frac{dI_k}{dS_k} \leq 0$ or $\varepsilon_k \leq 0$ is given by

$$
\begin{aligned}
1-\delta(S_k+\Theta) &> 0 \\
\delta(S_k+\Theta) &< 1. \\
\text{Since,}\quad 0 \leq \delta &\leq 2 \\
\text{Therefore,}\quad \hat{S}_k = S_k+\Theta &< \frac{1}{2}
\end{aligned} \tag{C.1}
$$

This reinforce that no single OOPE component can not dominate the total expenditure beyond a reasonable fraction.

Appendix D FGT-Inequality measure

This section provides the derivation of inequality measure in FGT type CHE as shown in Equation 11. Our objective is to find out the mean and variance of C_{ik} denoted by $\bar{C}_k$ and $\sigma^2(C_{ik})$ respectively.

$$\text{Note that, } C_{ik} = \frac{\upsilon_{ik}}{\hat{\alpha_k}} - 1 \quad \forall \upsilon_{ik} > \alpha_k \qquad i(1)H_k$$

$$\begin{aligned}
\bar{C}_k &= \frac{1}{H_k} \sum_{i=1}^{H_k} \left[\frac{\upsilon_{ik}}{\hat{\alpha_k}} - 1 \right] \\
\bar{C}_k &= \Gamma_k(1) \qquad \text{(D.1)} \\
\sigma^2 \left(C_{ik} \right) &= \frac{1}{H_k} \sum_{i}^{H_k} \left(C_{ik} - \bar{C}_k \right)^2 \\
&= \frac{1}{H_k} \sum_{i}^{H_k} \left(C_{ik} \right)^2 - \left(\bar{C}_k \right)^2 \\
&= \Gamma_k(2) - \Gamma_k^2(1) \\
\implies \Gamma_k(2) &= \Gamma_k^2(1) + \sigma^2(C_{ik}) \\
\text{Hence, } CHE_k(2) &= \left(\frac{H_k}{N} \right) \times \left[\Gamma_k^2(1) + \sigma^2(C_{ik}) \right] \qquad \text{(D.2)}
\end{aligned}$$

Equation D.2 expresses $CHE_k(2)$ in terms of incidence, intensity, and inequality. An alternative interpretation of the FGT-type CHE measure at $\lambda = 2$ is that it represents an incidence-weighted intensity-adjusted measure of inequality. This formulation enables the identification of the factors that drive the magnitude of the index. It also helps assess whether the overshoot distribution is dominated by many households just crossing the threshold or by a few households exceeding it by a large margin.

Appendix E Inverse rank weighted index threshold method: A hypothetical illustration

The hypothetical example of the inverse-rank weighted index (IRWI) method, illustrated in Table E.1, arranges twelve households by ascending per capita income (or consumption expenditure). It considers three healthcare expenditure components. For instance, household HH-1 allocates 0.234 of its total OOPE to component 1, with corresponding shares for other components shown in subsequent columns. The mean share of component 1 across all households is 0.291, and its concentration index (relative to per capita income) is 0.057. Using Equation 6, $\hat{W}_1$ is calculated as 0.275, while Equation 8 determines the catastrophic threshold for component 1 as 3.6%. Notably, HH-12 spend 6.25% (4000/64000) on component one of OOPE, surpassing the threshold and experiencing financial catastrophe.

Table E.1: Hypothetical Example of Inverse-Weighted Rank Method

	OOPE Components					OOPE Share			CHE Components Specific		
HH	Comp-1	Comp-2	Comp-3	OOPE	Income(pc)	s_{i1}	s_{i2}	s_{i3}	$H_{\hat{\alpha}_1}$	$H_{\hat{\alpha}_2}$	$H_{\hat{\alpha}_3}$
1	220	120	600	940	12000	0.234	0.128	0.638	0	0	1
2	460	100	900	1460	14000	0.315	0.068	0.616	0	0	1
3	0	0	0	0	17000				0	0	0
4	320	710	1200	2230	21000	0.143	0.318	0.538	0	0	1
5	600	860	1440	2900	25000	0.207	0.297	0.497	0	0	1
6	0	0	0	0	30000				0	0	0
7	0	0	0	0	35000				0	0	0
8	440	2200	300	2940	40400	0.150	0.748	0.102	0	1	0
9	8000	0	0	8000	46000	1.000	0.000	0.000	1	0	0
10	1200	5600	0	6800	50000	0.176	0.824	0.000	0	1	0
11	0	2200	1160	3360	58400	0.000	0.655	0.345	0	1	0
12	4600	5400	1600	11600	64000	0.397	0.466	0.138	1	1	0
$\hat{S}_k$						0.291	0.389	0.319			
CI_k						0.057	0.235	-0.338			
$\hat{W}_k$						0.275	0.298	0.427			
I_k						0.363	0.351	0.286			
$\hat{\alpha}_k$						3.6%	3.5%	2.9%			

Appendix F OOPE Components and Sub-Components

E.1. Doctor's/Surgeon's Fee: Total amount paid for doctor's/surgeon's fees during the hospital stay. The doctor(s) may or may not be attached to the hospital.

E.2. Medicines: Total amount paid for medicines (including drips) used during treatment, whether provided by the hospital or procured externally.

This includes recognized systems of medicines in India, regulated by the Department of AYUSH (*Ayurveda, Yoga & Naturopathy, Unani, Siddha, and Homeopathy*).

E.3. Diagnostic Tests: Total amount paid for diagnostic tests during hospital stay, regardless of where conducted.

E.4. Bed Charges: Amount paid for bed charges, including food if inseparable.

E.5. Other Medical Expenses: Includes all other treatment-related expenses:

- **E.5.1. Attendant Charges:** Charges for hired caregivers. Household members' services are not monetized.
- **E.5.2. Physiotherapy:** Charges for physiotherapy, regardless of provider affiliation.
- **E.5.3. Personal Medical Appliances:** Durable items like spectacles, pacemakers, crutches, etc.
- **E.5.4. Blood, Oxygen Cylinder, etc.:** Includes blood, oxygen, gloves, bandages, plaster, etc.
- **E.5.5. Other Medical Items:** Thermometers, blood pressure monitors, and similar items purchased by the household.

E.6. Medical Expenditure (Rs.): Total: Total of items 5 to 10 per admission case, ensuring inclusion of any package component.

E.7. Transport for Patient: Transport expenses for the patient to and from the hospital, including travel for diagnostic tests referred externally.

E.8. Other Non-Medical Expenses: Includes:

E.8.1. Food: Food costs for the patient, excluding home-provided meals.

E.8.2. Transport (Other than Ambulance): Transportation expenses for household members visiting or assisting the patient.

E.8.3. Lodging Charges of Escorts: Lodging costs for escorts during the hospital stay.

E.8.4. Other Expenses: Incidental expenses like phone charges, toiletries for patient and escorts.

Appendix G Component specific thresholds and CHE pattern-71st round

Table G.1: Descriptive Statistics

Components	Incidence	Mean (INR)	Std. Err.	$\mu \pm 1.96\frac{\sigma}{\sqrt{n}}$ L	$\mu \pm 1.96\frac{\sigma}{\sqrt{n}}$ U	N
Doctor's/ surgeon's fee	55.53%	6931	130	6676	7187	21409
Medicines	82.52%	6070	92	5890	6251	34102
Diagonistic tests	67.03%	3004	47	2912	3095	27723
Bed charges	52.18%	4170	85	4004	4336	20496
Other medical expenses	58.41%	2915	76	2765	3065	23801
Transport for patient	89.40%	787	8	771	804	36236
Other non-medical expenses	91.52%	1594	17	1562	1627	36979
Total OOPE: IP	99.53%	16993	222	16558	17428	40107
Doctor's/ surgeon's fee	46.65%	5401	236	4939	5863	11037
Medicines : AYUSH	7.76%	10597	473	9669	11525	1729
Medicines	81.92%	14510	190	14137	14883	19736
Diagonistic tests	16.79%	11994	283	11439	12549	4439
Other medical expenses	9.20%	7626	363	6914	8337	2406
Transport for patient	49.46%	3103	88	2930	3275	12019
Other non-medical expenses	27.21%	3975	119	3741	4209	6540
Total OOPE: OP	93.61%	21963	320	21337	22590	22280
Consumption Expenditure	100.00%	87996	267	87474	88519	65922

Table G.2: Comparison of CHE Incidence and Intensity of OOPE Components Between Conventional and Proportional Methods

	Wagstaff Method				Atagabu Method				
**Components**	H_{10}	CI_{10}	MPG_{10}	$CI_{M_{10}}$	α_k	H_{α_k}	CI_{α_k}	MPG_{α_k}	$CI_{M_{\alpha_k}}$
**Hospitalisation Cases**									
Doctor's/Surgeon's Fee	1.53%	0.131	17.44%	-0.072	2.28%	4.53%	0.132	10.35%	-0.039
Medicines	2.11%	-0.024	17.62%	-0.084	2.96%	6.46%	0.001	9.76%	-0.066
Diagnostic Tests	0.68%	0.074	12.87%	-0.042	1.29%	5.23%	0.117	4.92%	-0.036
Bed Charges	0.56%	0.025	14.35%	-0.136	1.19%	6.07%	0.053	4.03%	-0.054
Other Medical Expenses	0.51%	0.120	18.86%	0.005	1.01%	4.40%	0.041	4.97%	0.053
Transport for Patient	0.10%	-0.303	8.99%	-0.153	0.42%	7.80%	-0.101	1.29%	-0.090
Other Non-Medical Expenses	0.35%	-0.167	8.85%	-0.018	0.86%	7.53%	-0.068	2.58%	-0.047
**Outpatient Cases**									
Doctor's/Surgeon's Fee	2.08%	-0.084	15.14%	-0.074	1.23%	13.45%	0.052	6.16%	-0.122
Medicines: AYUSH	0.85%	-0.044	32.26%	-0.352	0.40%	2.44%	0.047	16.86%	-0.295
Medicines	12.44%	-0.033	24.67%	-0.128	5.78%	17.40%	-0.008	21.26%	-0.129
Diagnostic Tests	2.10%	-0.088	23.32%	-0.054	0.98%	5.26%	0.093	15.07%	-0.173
Other Medical Expenses	0.67%	-0.075	23.06%	-0.172	0.34%	2.70%	0.034	10.48%	-0.167
Transport for Patient	1.20%	-0.280	14.54%	0.034	0.75%	13.16%	-0.020	4.17%	-0.140
Other Non-Medical Expenses	1.08%	-0.297	15.94%	-0.025	0.53%	8.37%	-0.069	5.59%	-0.158

Table G.3: Rank weighted component wise catastrophic threshold and headcount ratio

Components	$\hat{S}_k$	CI_k	$\hat{W}_k$	I_k	$\hat{\alpha}_k$	$H_{\hat{\alpha}_k}$
**Hospitalisation cases**						
Doctor's/ surgeon's fee	0.123	0.172	0.102	0.150	1.50%	5.40%
Medicines	0.295	-0.033	0.304	0.116	1.16%	10.19%
Diagnostic tests	0.104	0.081	0.095	0.151	1.51%	4.77%
Bed charges	0.080	0.181	0.066	0.156	1.56%	5.17%
Other medical expenses	0.076	0.030	0.073	0.154	1.54%	3.37%
Transport for patient	0.118	-0.138	0.134	0.144	1.44%	2.60%
Other non-medical expenses	0.205	-0.098	0.225	0.129	1.29%	5.81%
**Outpatient cases**						
Doctor's/ surgeon's fee	0.117	0.036	0.113	0.148	1.48%	12.72%
Medicines : AYUSH	0.048	0.004	0.048	0.159	1.59%	1.94%
Medicines	0.625	0.002	0.624	0.063	0.63%	26.38%
Diagnostic tests	0.042	0.074	0.039	0.160	1.60%	4.91%
Other medical expenses	0.018	-0.031	0.018	0.164	1.64%	2.07%
Transport for patient	0.097	-0.015	0.099	0.150	1.50%	9.34%
Other non-medical expenses	0.052	-0.124	0.059	0.157	1.57%	6.17%

Table G.4: Catastrophic Headcount at the aggregate

Item	Threshold	Headcount
Aggregate IP OOPE with 10% threshold	10%	6.63%
At least one IP components is catastrophic	Proportionate Methodd	12.39%
	Inverse Rank Weight threshold	12..07%
Aggregate OP OOPE with 10% threshold	10%	17.30%
At least one OP components is catastrophic	Proportinate method	25.42%
	Inverse Rank Weight threshold	29.12%

Table G.5: FGT based incidence, intensity and inequality measures

Components	$\hat{\alpha}_k$	$CHE(\lambda)$ λ=0	λ=1	λ=2	$\Gamma_k(\lambda)$ λ=1	λ=2	$\Gamma_k^2(1)$	$\sigma^2(v_{ik}^*)$
**Hospitalisation cases**								
Doctor's/ surgeon's fee	1.50%	0.054	0.339	16.1	6.276	297.5	39.4	258.2
Medicines	1.16%	0.102	0.670	37.6	6.574	368.4	43.2	325.2
Diagonistic tests	1.56%	0.048	0.157	3.6	3.282	75.6	10.8	64.8
Bed charges	1.51%	0.052	0.151	4.8	2.911	92.2	8.5	83.7
Other medical expenses	1.54%	0.034	0.128	5.7	3.806	170.4	14.5	155.9
Transport for patient	1.44%	0.026	0.038	0.3	1.472	10.5	2.2	8.3
Other non-medical expenses	1.29%	0.058	0.128	1.2	2.211	20.8	4.9	15.9
**Outpatient cases**								
Doctor's/ surgeon's fee	1.48%	0.127	0.54	49.1	4.2	386.2	17.9	368.4
Medicines : AYUSH	1.59%	0.019	0.24	32.6	12.5	1683.3	156.8	1527.6
Medicines	0.63%	0.264	7.69	1252.8	29.2	4749.3	850.2	3899.4
Diagonistic tests	1.60%	0.049	0.48	20.5	9.7	418.3	93.8	324.6
Other medical expenses	1.64%	0.021	0.15	7.3	7.4	354.6	55.3	299.4
Transport for patient	1.50%	0.093	0.31	8.6	3.3	91.8	11.0	80.9
Other non-medical expenses	1.57%	0.062	0.25	5.6	4.1	91.1	16.5	74.7

References

Wagstaff, A., & van Doorslaerc, E. (2003). Catastrophe and impoverishment in paying for health care: With applications to vietnam 1993-98. *Health Economics*, *12*(11), 921–34.
